# Motion-Tolerant Non-Contact Heart-Rate Measurements from Radar Sensor Fusion

**DOI:** 10.3390/s21051774

**Published:** 2021-03-04

**Authors:** Yu Rong, Arindam Dutta, Alex Chiriyath, Daniel W. Bliss

**Affiliations:** Center for Wireless Information Systems and Computational Architectures (WISCA), School of Electrical, Computer and Energy Engineering, Arizona State University, Tempe, AZ 85281, USA; adutta7@asu.edu (A.D.); achiriya@asu.edu (A.C.); d.w.bliss@asu.edu (D.W.B.)

**Keywords:** radar, vital signs, random body movement cancellation, UWB, privacy preserving

## Abstract

Microwave radar technology is very attractive for ubiquitous short-range health monitoring due to its non-contact, see-through, privacy-preserving and safe features compared to the competing remote technologies such as optics. The possibility of radar-based approaches for breathing and cardiac sensing was demonstrated a few decades ago. However, investigation regarding the robustness of radar-based vital-sign monitoring (VSM) is not available in the current radar literature. In this paper, we aim to close this gap by presenting an extensive experimental study of vital-sign radar approach. We consider diversity in test subjects, fitness levels, poses/postures, and, more importantly, random body movement (RBM) in the study. We discuss some new insights that lead to robust radar heart-rate (HR) measurements. A novel active motion cancellation signal-processing technique is introduced, exploiting dual ultra-wideband (UWB) radar system for motion-tolerant HR measurements. Additionally, we propose a spectral pruning routine to enhance HR estimation performance. We validate the proposed method theoretically and experimentally. Totally, we record and analyze about 3500 s of radar measurements from multiple human subjects.

## 1. Introduction

Vital-sign monitoring (VSM) devices are extremely important for human healthcare and wellness, whether it be consumer-grade devices that promote self-health monitoring or medical-grade devices that aid in early diagnosis and facilitate treatment. Remote sensing using radar is one of such technologies that supports non-contact vital-sign measurements [1,2,3]. Microwave ultra-wideband (UWB) radar systems have good penetrative capability and range resolution, which enables them to non-invasively monitor internal physiological motion of the organs of a body, such as the heart or lungs, by transmitting low-energy electromagnetic waves. Thus, such radar systems can extract the heart-rate (HR) and breathing rate (BR) of a subject remotely from a distance.

Radar-based health monitoring devices have a myriad of potential applications, such as infant monitoring [4], sleep monitoring [5,6,7], elder care [8], and animal care [9]. The non-contact feature of radar makes it extremely useful for healthcare applications, such as remote patient monitoring, and enabling a more comfortable and efficient caregiving. A non-contact way of measuring vital signs decreases the risk of infection for healthcare professionals, and thus reduces the risk of any contagious virus transmission. This cannot only be beneficial in the current coronavirus disease 2019 pandemic scenario [10], but also in the long run for general patient monitoring and rehabilitation care.

Recently, there has been a strong increase in demand for consumer-grade and medical-grade VSM devices. A significant portion of current vital-sign sensors are either wearable-based or use optical sensors such as cameras. These approaches have drawbacks such as requiring constant contact with the subject or a lack of privacy [11]. The optical sensors based on remote imaging photoplethysmography (PPG) [12] are a competing non-contact HR monitoring technology for radar approach. HR can be extracted using a low-cost color camera, such as a webcam, by processing a sequence of recorded video images. However, the camera approach is subjective to skin-tones [13], motion artifacts [14,15] and lighting condition [16,17]. Accordingly, significant efforts are investigated on imaging-based robust methods for PPG, such as algorithmic development [18,19,20] and exploration of non-visible light waves [21]. Besides raising privacy issues, the most obvious operational disadvantage of remote imaging PPG measurement is that optical sensors in general have poor penetrative capability. They do not penetrate clothes and blanket, which is required for non-interruptive sensing for continuous, long-term monitoring. Thus, optical sensors are limited for measurements from light-of-sight body spots and directly exposed skin areas, such as hand and face. Alternatively, microwave radar technology presents a very attractive option for ubiquitous short-range VSM due to its non-contact, see-through, and privacy-preserving features. Vast frequency bands have been explored using radar electronics for VSM, ranging from a few gigahertz (GHz) in low frequency band [1], tens of GHz in millimeter wave band [22,23], and hundreds of GHz in Terahertz wave band [24].

To date, most work on non-contact radar-based VSM sensors has focused on monitoring a single human stationary subject. Multiple human subjects in ideal situation were considered in these works [25,26,27,28]. Often, the subjects would typically be instructed to remain motionless by seating still or lying down, and would be in a quiet and controlled environment. This is because a small random body motion (a few centimeters) is often stronger than breathing-induced (millimeter to centimeter) and heartbeat-induced (much less than millimeter) physiological motions, potentially overwhelming the desired signal. A few attempts [29,30,31,32,33] have been made to cope with random body movement (RBM) noise via differential measurement/processing, such as multiple radars at different body sides [30], radar and camera fusion [33], multi-frequency radar systems [34], sensing system exploiting auxiliary sensors attached to the human body [32]. In particular, in reference [29] two continuous wave radars were placed on opposite sides of a person to cancel body movement through differential detection.

In this paper, we focus on investigation of motion-tolerant radar method for HR estimation in realistic settings with a dual-UWB impulse radar system. The two UWB radar sensors are strategically placed at the front side of human chest to create differential measurement of a common chest motion and upper body motion, for example, back-and-forth motion (BFM). The UWB sensor towards the left chest captures major heartbeat signal plus other common signals present in the other sensor on the right side of the chest. The directly sampled radio frequency (RF) signals are fused and converted in the complex baseband for spectral analysis. The recovery of useful vital-sign spectral structure by RF differencing operation to suppress BFM and breathing motion is theoretically and experimentally justified. Subsequently, a spectral-based HR pruning technique is proposed to exploit spectral heartbeat harmonics [26,35] when the fundamental heartbeat is masked by motion residual. The proposed method is tested against comprehensive motion-tolerant experiments. Finally, the impact of breathing dynamics and heartbeat dynamics on HR estimation performance are thoroughly discussed.

## 2. Materials and Methods

### 2.1. Experimental Setup and Design

The study was approved by the ethics committee of the Arizona State University (approval number: HPR-5-3b). All research was performed in accordance with relevant guidelines and regulations. Three human subjects were included during the study. In particular, the studied subjects have different physical conditions. One evidence is that some subjects have relatively lower resting HR while other subjects relatively higher resting HR. One of the goals of this experimental study is to demonstrate the limitations of conventional radar approaches. The existing methods mostly assume that (1) the testing subjects are stationary and (2) spectral separation is enough for HR calculation. These assumptions are in general not true. These facts are demonstrated through multiple relatively longer-term measurements, which capture involuntary RBM in the prolongated recordings. Additionally, multiple challenging poses are considered. These include stable seating (with back support), free-style seating (without back support), and standing. The second goal of this study is to provide a motion-tolerant radar method with active motion cancellation technique to solve the aforementioned issues. For validation, the proposed algorithm is tested in several carefully designed experiments, in which the test subjects are instructed to move back and forth constantly.

**Figure 1:**
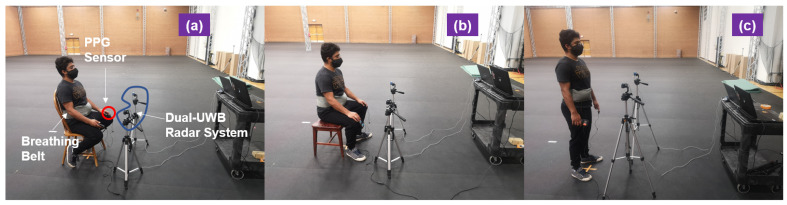
Novel dual-UWB radar system same-side scenario. (**a**) Stable seating; (**b**) free-style seating; (**c**) standing.

A novel dual-radar system setup is presented as illustrated in Figure 1. Both radars are placed at chest height in the front of the human body but with a tilted angle, 45°, with respect to a center line which is perpendicular to the chest plane. Exploiting spatial diversity with this frontal side dual-radar setup, similar radar channels can be created for RBM cancellation purpose, achieving the goal of improved vital-sign detection. The sensor pointing to the left chest area where the heart chamber is located captures all kinds of body motion including chest motion and heartbeat. As for the other sensor pointing to the right side of the chest, it captures the common body motion, as seen in another radar channel except that it only sees a small portion of heartbeat signal. Due to channel noise, the weak heartbeat signal is negligible in this sensor. Thus, the following assumption is made here that heartbeat signal is only present in one radar channel while the rest of body motions are common to both radar channels.

### 2.2. Measurement Devices

**Figure 2:**
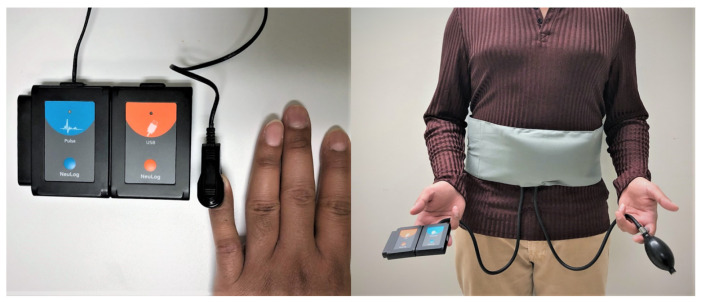
Neulog optical pulse sensor and respiration monitor belt logger sensor used to record PPG signals and breathing signals from the test subject as standard reference for heart-rate and breathing rate.

A fingertip oximeter together with a digital dataloger (NeuLog heart-rate and pulse logger sensor, model NUL-208) was used to collect standard PPG signal as pulse reference from the test subjects. The PPG signal is acquired at a sampling rate of 50 Hz. A chest air pressure belt (NeuLog respiration monitor belt logger sensor, model NUL-236) was used for providing breathing reference. The breathing signal is also acquired at a sampling rate of 50 Hz. The pulse reference device and breathing reference device are synchronized in hardware (Figure 2).

The dual-UWB radar system (Figure 1) consists of two independent radar sensors (Xethru X4M03 development kit) equal distant from the test subject. These radar sensors operate at center frequency 7.3 GHz with effective signal bandwidth 1.4 GHz. On board patch antennas have directional beamwidth about 60° in azimuth and elevation. Each of these sensors employs an UWB impulse signaling scheme and direct RF receiver architecture [36], critical sampling at RF and preserving all the information, including vital-sign signals, carried in the received signal in the digital domain [37]. For spectral masking, the transmitter employs a biphase coding modulation scheme, where the encoding bit sequence is independently generated at each sensor node using a pseudo-random noise pattern [36]. Thus, each sensor only sees its own signal backscattered from the target of interest. The two sensors are physically wired to a control unit and synchronized in time.

### 2.3. Proposed Signal Processing

**Figure 3:**
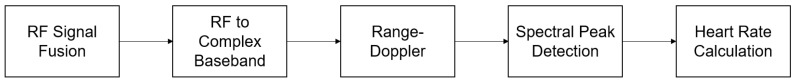
Block diagram showing the main processing steps to extract human subject’s heart-rate from radar signals.

The major signal-processing steps are highlighted in Figure 3 to extract human subject’s HR. The proposed signal-processing algorithm can be categorized into five processing blocks, including direct RF signal fusion, RF to complex baseband conversion, range-Doppler analysis to locate the range of interest, spectral peak detection and HR calculation. Each processing block is discussed in the following sections.

#### 2.3.1. Active Motion Cancellation

#### Direct RF Signal Processing

Active motion cancellation is a key processing setup before any conventional signal processing is applied. With explicit suppression of any large RBMs, long-term heart-rate monitoring is feasible because no human subject can maintain an absolute stationary position for an extended period of time in any relaxed state. Two radar channels provide two slightly different observations from the two strategically positions for sensor fusion operation. The ability to access directly sampled RF samples allows mixing the two copies of RF radar scans (left side $S_{L}^{RF}$ and right side $S_{R}^{RF}$) at the same time. The differential measurement by differencing the two RF radar scans (left side $S_{L}^{RF}$ and right side $S_{R}^{RF}$) provides a desirable residual signal with enhanced pulse signal, such that the motion artifact and stronger breathing is suppressed prior to apply other signal-processing techniques. The received RF signal is modeled as a nonlinear function (£{}) of normalized RBM R(τ,t), breathing signal B(τ,t) and pulse signal H(τ,t) and receiver noise W(τ,t). These components are organized in two-dimensional (2-D) matrix format, as follows,

(1) $$\begin{array}{cc} S_{L}^{RF}\left(\tau ,t\right) & =£\left\{R\left(\tau ,t\right),B\left(\tau ,t\right),H\left(\tau ,t\right),W_{L}\left(\tau ,t\right)\right\} \end{array}$$

(2) $$\begin{array}{cc} S_{R}^{RF}\left(\tau ,t\right) & =£\left\{R\left(\tau ,t\right),B\left(\tau ,t\right),W_{R}\left(\tau ,t\right)\right\}, \end{array}$$

where τ denotes fast-time samples corresponding to the range domain while *t* denotes slow-time samples corresponding to the temporal domain. The HR calculation is operated along the slow-time samples. By differencing Equations (1) and (2) at $t=t_{i}$, where *i* denotes *i*th slow-time index, we have,

(3) $$\begin{array}{c} \begin{array}{c} s_{Diff}^{RF}\left(\tau ,t_{i}\right)=s_{L}^{RF}\left(\tau ,t_{i}\right)-s_{R}^{RF}\left(\tau ,t_{i}\right) \end{array}. \end{array}$$

#### Baseband Signal Processing

The residual RF signal Equation (3) is digitally downconverted to complex baseband by mixing with a nominal carrier.

(4) $$\begin{array}{c} s_{Diff}^{BB}\left(\tau_{j},t\right)=s_{Diff}^{RF}\left(\tau_{j},t\right)e^{-j2\pi f_{c}\tau }. \end{array}$$

The common approach is to extract the Doppler phase component from Equation (4), using $p_{Diff}^{BB}\left(\tau_{j},t\right)=\mathrm{unwrap}\;\;\left[\;\mathrm{arctangent}\;\;[s^{BB}\left(\tau_{j},t\right)]\right]$. “arctangent” denotes arctangent demodulation operating on the ratio of real component and imaginary component of the baseband signal $s^{BB}\left(\tau_{j},t\right)$ at the range of interest $d_{j}=2\;c\;\tau_{j}$. Moreover, “unwrap” operator is applied to deal with the wrapping problem when the absolute jumps between consecutive phase samples are greater than or equal to 180°.

However, it is found that the phase-based method does generate stable spectrum over time in the presence of RBMs in this study. Instead of phase extraction, the spectral peak detection for HR calculation is performed on the complex signals (Equation (4)) because the higher-order spectral features of heartbeat in complex signal frequency domain aid in HR calculation when the residual motion interference masks the fundamental heartbeat spectral energy.

#### 2.3.2. Analytical Spectral Analysis of Fused Sensor Signal

The spectral representation of the complex-based signal $s_{Diff}^{BB}\left(\tau_{j},t\right)$ is derived to demonstrate that (1) the major frequency components related BFM are suppressed via differencing RF signals and (2) the heartbeat harmonic spectral features are maintained in the complex-based signal model and thus is helpful for rate calculation. For convenience, a simplified motion model is used for characterizing the RBM profile,

(5) $$R\left(t\right)=A_{R}\;\;\sin\left(2\pi f_{R}t\right),$$

which matches the constant BFM considered in this study. $A_{R}$ is the amplitude of BFM and $f_{R}$ denotes the cyclic frequency of the activity. Similarly, the respiratory activity and the cardiac activity are defined using the same model but with appropriate amplitude values and frequency values to match their physical characteristics.

(6) $$\begin{array}{cc} B(t) & =A_{B}\;\;\sin\left(2\pi f_{B}t\right) \end{array}$$

(7) $$\begin{array}{cc} H(t) & =A_{H}\;\;\sin\left(2\pi f_{H}t\right), \end{array}$$

where the amplitude values of these three activities follow $A_{R}\gg A_{B}\gg A_{H}$, $f_{B}$ for normal human subject is around 10 to 20 beats per minute (BPM) and $f_{H}$ 60 to 100 BPM. For UWB impulse radar, the received pulse is modeled as an attenuated and shifted version of the transmitted pulse p(τ). The BFM, breathing motion and heartbeat motion modulate the pulse and create a time-varying delay profile $\tau_{D}\left(t\right)$ around a nominal distance $d_{0}$ with the associated time-delay $\tau_{0}=2d_{0}/c$. The backscattered signal from the left chest is given as,

(8) $$\begin{array}{cc} s_{L}^{RF}\left(\tau ,t\right) & =A_{T}\;\;p\left(\tau -\tau_{D,L}\left(t\right)\right) \end{array}$$

(9) $$\begin{array}{cc} \tau_{D,L}\left(t\right) & =\frac{2\left(d_{0}+R\left(t\right)+B\left(t\right)+H\left(t\right)\right)}{c}, \end{array}$$

where $A_{T}$ denotes the amplitude of the target response and *c* speed of light. The backscattered signal from the right chest is given as,

(10) $$\begin{array}{cc} s_{R}^{RF}\left(\tau ,t\right) & =A_{T}\;\;p\left(\tau -\tau_{D,R}\left(t\right)\right) \end{array}$$

(11) $$\begin{array}{cc} \tau_{D,R}\left(t\right) & =\frac{2\left(d_{0}+R\left(t\right)+B\left(t\right)\right)}{c}. \end{array}$$

Accordingly, the differential complex baseband signal $s_{Diff}^{BB}\left(\tau_{j},t\right)$ is obtained,

(12)sDiffBB(τ,t)=sLBB(τ,t)−sRBB(τ,t)(13)=ATp(τ−τD,L(t))−p(τ−τD,R(t))e−j2πfcτ.

For convenience, the $s_{L}^{BB}\left(\tau ,t\right)$ and $s_{R}^{BB}\left(\tau ,t\right)$ in Equation (12) are evaluated separately. Through forward and backward Fourier transforms (FT) with respect to *t* and τ, the FT of $s_{L}^{BB}\left(\tau ,t\right)$ is obtained as (see Appendix A for detailed derivation of $s_{L}^{BB}\left(\tau ,f\right)$),

(14)sLBB(τ,f)=AT∑k=−∞∞∑l=−∞∞∑q=−∞∞δ(f−kfR−lfB−qfH)×∫−∞∞dν[P(ν)ej2πν(τ−τ0)   ×Jk4πνARcJl4πνABcJq4πνAHc](15)    =AT∑k=−∞∞∑l=−∞∞∑q=−∞∞CL,(k,l,q)(τ)δ(f−kfR−lfB−qfH),

where $J_{k}\left(.\right)$ denotes the Bessel function of the first kind [38] and $C_{L,(k,l,q)}\left(\tau \right)$ is given as the following equation and its absolute value achieves the maximum at $\tau_{0}$,

(16) $$C_{L,(k,l,q)}\left(\tau \right)=\int_{-\infty }^{\infty }d\nu \;\;[P\left(\nu \right)\;\;e^{j2\pi \nu (\tau -\tau_{0})}\times J_{k}\left(4\pi \nu \frac{A_{R}}{c}\right)\;\;J_{l}\left(4\pi \nu \frac{A_{B}}{c})J_{q}\left(4\pi \nu \frac{A_{H}}{c}\right)\right],$$

where P(ν) denotes the FT of the transmitted pulse p(τ). Then,

(17) $$\begin{array}{cc} \left|s_{L}^{BB}\left(\tau ,f\right)\right| & \leq |A_{T}|\;\sum_{k=-\infty }^{\infty }\sum_{l=-\infty }^{\infty }\sum_{q=-\infty }^{\infty }|C_{L,(k,l,q)}\left(\tau_{0}\right)|\delta \left(f-kf_{R}-lf_{B}-qf_{H}\right)=|s_{L}^{BB}\left(\tau_{0},f\right)|. \end{array}$$

On the other hand, the FT of $s_{R}^{BB}\left(\tau_{j},t\right)$ with respect to *t* is derived in the same fashion,

(18) $$\begin{array}{cc} s_{R}^{BB}\left(\tau ,f\right) & =A_{T}\;e^{-j2\pi f_{c}\tau_{0}}\sum_{k=-\infty }^{\infty }\sum_{l=-\infty }^{\infty }C_{R,(k,l)}\left(\tau \right)\delta \left(f-kf_{R}-lf_{B}\right), \end{array}$$

(19) $$\begin{array}{cc} |s_{R}^{BB}\left(\tau ,f\right)| & \leq \;|A_{T}|\sum_{k=-\infty }^{\infty }\sum_{l=-\infty }^{\infty }|C_{R,(k,l)}\left(\tau_{0}\right)|\;\;\delta \left(f-kf_{R}-lf_{B}\right)=\left|s_{R}^{BB}\left(\tau_{0},f\right)\right|, \end{array}$$

where $C_{R,(k,l)}\left(\tau \right)$ is given as,

(20) $$C_{R,(k,l)}\left(\tau \right)=\int_{-\infty }^{\infty }d\nu \;\;[P\left(\nu \right)\;\;e^{j2\pi \nu (\tau -\tau_{0})}\times J_{k}\left(4\pi \nu \frac{A_{R}}{c}\right)\;\;J_{l}\left(4\pi \nu \frac{A_{B}}{c})\right].$$

By inspecting Equations (12), (15) and (19), the FT of $s_{Diff}^{BB}\left(\tau_{j},t\right)$ with respect to *t* is approximated as,

(21) $$\begin{array}{cc} \left|s_{Diff}^{BB}\left(\tau_{0},f\right)\right| & \approx \;|A_{T}|\;\sum_{k=-\infty }^{\infty }\sum_{l=-\infty }^{\infty }\sum_{q=-\infty }^{\infty }|C_{L,(k,l,q)}\left(\tau_{0}\right)|\delta \left(f-kf_{R}-lf_{B}-qf_{H}\right)\\ & -|A_{T}|\sum_{k=-\infty }^{\infty }\sum_{l=-\infty }^{\infty }\left|C_{R,(k,l)}\left(\tau_{0}\right)\right|\;\;\delta \left(f-kf_{R}-lf_{B}\right). \end{array}$$

The theoretical spectral power representation of the complex baseband signal $s_{Diff}^{BB}\left(\tau_{0},t\right)$ consists of weighted impulse pulse train located at frequency locations from combinations of $f_{R},f_{B}$ and $f_{H}$. To focus on the most relevant spectral harmonics, only consider the orders of harmonics up to the second order in the analytical analysis and consider the positive frequency locations given the spectral symmetry in Equation (21), meaning that k,l,q is either 0 or ±1 or 2 and |k|+|l|+|q|≤2. The corresponding weights $|C_{L,(k,l,q)}|$ and $|C_{R,(k,l)}|$ significantly decrease when the order of harmonics increases (>2). Without loss of generality, let $f_{R}<f_{B}<f_{H}$. Therefore, Equation (21) is expanded out as,

(22)|sDiffBB(τ0,f)| ≈ |AT|{|CL,(1,0,0)(τ0)|δ(f−fR)+ |CL,(0,1,0)(τ0)|δ(f−fB)+|CL,(0,0,1)(τ0)|δ(f−fH)+|CL,(2,0,0)(τ0)|δ(f−2fR)+ |CL,(0,2,0)(τ0)|δ(f−2fB)+|CL,(0,0,2)(τ0)|δ(f−2fH)+|CL,(1,−1,0)(τ0)|δ(f+fR−fB)+|CL,(0,−1,1)(τ0)|δ(f+fB−fH)+|CL,(−1,0,1)(τ0)|δ(f+fR−fH)+|CL,(1,1,0)(τ0)|δ(f−fR−fB)+|CL,(0,1,1)(τ0)|δ(f−fB−fH)+|CL,(1,0,1)(τ0)|δ(f−fR−fH)}−|AT|{|CR,(1,0)(τ0)|δ(f−fR)+|CR,(0,1)(τ0)|δ(f−fB)+|CR,(2,0)(τ0)|δ(f−2fR)+|CR,(0,2)(τ0)|δ(f−2fB)+|CR,(−1,1)(τ0)|δ(f+fR−fB)+|CR,(1,1)(τ0)|δ(f−fR−fB)}(23)≈AT{|CL,(1,0,0)(τ0)|δ(f−fH)+|CL,(2,0,0)(τ0)|δ(f−2fH)+|CL,(0,−1,1)(τ0)|δ(f+fB−fH)+|CL,(−1,0,1)(τ0)|δ(f+fR−fH)+|CL,(0,1,1)(τ0)|δ(f−fB−fH)+|CL,(1,0,1)(τ0)|δ(f−fR−fH)}.

The result in Equation (23) is important since it implies that BFM R(t) and breathing motion B(t) is suppressed in the spectral domain and the dominant spectral components are the heartbeat harmonics. The last four terms in Equation (23) representing frequency intermodulations around the fundamental heartbeat frequency, but will not cause ambiguity because the weight of fundamental heartbeat is stronger than the other weights ($|C_{L,(1,0,0)}\left|>\right|C_{L,(0,-1,1)}\left|,\right|C_{L,(-1,0,1)}\left|,\right|C_{L,(0,1,1)}|$ and $|C_{L,(1,0,1)}|$) and more importantly the second-order harmonic of heartbeat ($2f_{H}$) further way from these intermodulations interference provides additional trace of HR. From Equation (22) to Equation (23), the weighted impulse responses at the common frequencies are cancelled out because of the following assumption,

(24) $$C_{L,(k,l,0)}\left(\tau_{0}\right)\approx C_{R,(k,l)}\left(\tau_{0}\right).$$

This approximation is obtained by invoking the mean value theorem from Equation (16),

(25)CL,(p,q,0)(τ0)≈Δf×P(fc)Jk4πfcARcJl(4πfcABc)J04πfcAHc(26)≈Δf×P(fc)Jk4πfcARcJl4πfcARc=CR,(p,q)(τ0),

where Δf represents the signal bandwidth. The last term in Equation (25), $J_{0}(4\pi f_{c}A_{H}/c)\approx 1$. Given the radar system parameter $f_{c}=7.3$ GHz and $A_{H}=$ 0.08 mm [39], $J_{0}(4\pi f_{c}A_{H}/c)$ gives a value of 0.99986.

### 2.4. Spectral-Based Heart-Rate Calculation Algorithm

**Figure 4:**
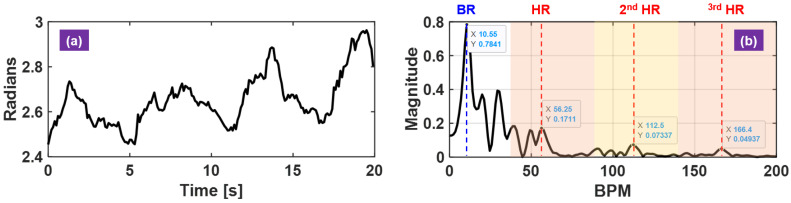
Spectral-based heart-rate (HR) and breathing rate (BR) calculation. (**a**) raw Doppler phase from single radar measurement dominated by breathing motion; (**b**) the corresponding complex vital-sign spectrum with the most relevant spectral features labeled with help of breathing and pulse references. The shaped areas denote the frequency region of the fundamental HR and HR harmonics.

It is known that HR calculation from spectral peak selection in radar-based approaches suffers from overwhelming interference of breathing harmonics [35]. One typical such radar measurement is shown in Figure 4. The fundamental HR location overlaps with the higher-order breathing harmonic (5th in this example), which makes it challenging to calculate HR over time without external reference (Figure 4b). Therefore, the heartbeat spectral harmonic features are exploited to improve the detection robustness and help to identify the fundamental HR.

**Figure 5:**
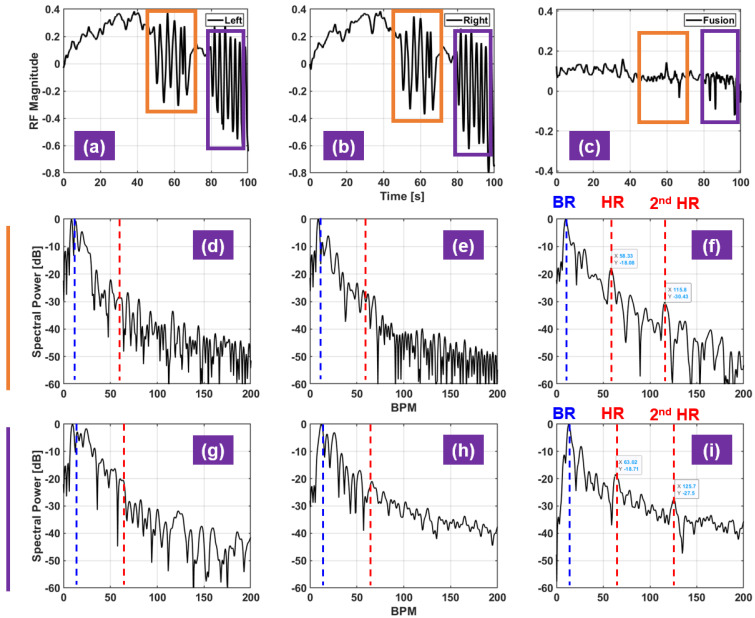
Heart-rate (HR) and breathing rate (BR) recovery in the presence of BFMs using RF fusion technique. From 45 to 72-s and 80 to 100 s, the human subject in free-style sitting position was instructed to move back and forth constantly. (**a**,**b**) represent the RF signals from the sensor pointing to the left side of the chest and to the right side of the chest; (**c**) represents the differential RF signal; while (**d**–**f**) show the corresponding vital-sign spectra from complex baseband signals, before and after motion cancellation, using 20-s of data from the highlighted time interval in (**a**–**c**) from 45 to 72-s; (**g**–**i**) show the corresponding vital-sign spectra from complex baseband signals, before and after motion cancellation, using 20-s of data from the highlighted time interval in (**a**–**c**) from 78 to 100-s.

When RBM is not present, the received signal is dominated by the breathing signal and the pulse signal is often found at least 10-dB to 20-dB weaker than the breathing signal. The pulse signal in the temporal waveform is overwhelmed as seen in Figure 4a, and thus the spectral-based peak detection algorithm is commonly used. However, herein the scenario of interest is HR recovery when constant RBM occurs (Figure 5a,b). Directly applying conventional signal processing renders failure without explicit motion cancellation (Figure 5d,e). On the other hand, the proposed HR calculation is operated on RBM suppressed input signals, it is possible to reuse these existing signal-processing steps for rate calculation, as demonstrated in Figure 5f.

#### Spectral Peak Pruning Routine

Meaningful vital-sign spectrum is revealed with the aforementioned active motion cancellation technique (Figure 5f) when BFM occurs. Afterwards, a spectral peak pruning routine is proposed to provide consistent HR estimate. Based on common knowledge, a heartbeat bandpass filter is first applied. Please note that this frequency range should be tuned matching the testing human subject’s resting HR. Then, this range is divided into *N* equally spaced intervals to recover the fundamental HR and up to the *N*th heartbeat harmonic. Within each frequency interval, the *C* most significant peaks are recorded. The *c*th peak location in the *n*th frequency interval, $Loc_{c}^{N}$, is normalized relative to the order of harmonic *n*, and *n* = 1, …, *N*. This is because the HR harmonics are multiple of the fundamental HR.

(27) $$e_{c}^{n}=\frac{{Loc}_{c}^{n}}{n},$$

where *c* denotes the *c*th largest peak and *c* = 1, …, *C*. A set E of potential HR estimates is obtained,

(28) $$\hat{hr}\in E=\left\{e_{1}^{1},\ldots ,e_{c}^{1},\ldots ,e_{C}^{1},\;|\;\ldots \;\ldots \;|,\;e_{1}^{n},\ldots ,e_{c}^{n},\ldots ,e_{C}^{n},\;|\;\ldots \;\ldots \;|,\;e_{1}^{N},\ldots ,e_{c}^{N},\ldots ,e_{C}^{N}\right\}.$$

The final HR estimate is chosen from the set E using a majority vote strategy. The HR estimate is calculated as,

(29) $$\hat{hr}=\{\mathrm{mean}[\sum_{n_{*}}e_{c_{n_{*}}}^{n_{*}}];\;\;\underset{n_{*}}{\mathrm{argmax}}\;\;\lambda \},$$

where $n_{*}$ belongs to a subgroup from n=1,…,N such that they ($e_{c_{n_{*}}}^{n_{*}}$) yields the most common votes λ, where 0≤λ≤N2. A common vote is achieved when,

(30) $$|e_{c_{1}}^{n_{1}}-e_{c_{2}}^{n_{2}}|\;<=\eta ,\;\;\;\;\mathrm{when}\;\;n_{1}\neq n_{2},$$

where $c_{1}$ or $c_{2}$ = 1, …, *C* and $n_{1}$ or $n_{1}$ = 1, …, *N*. η is a consensus tolerance parameter and is set to 2 BPM empirically. If no consensus (λ = 0) is found using Equation (30) in the set E, then the testing estimate that has smallest difference to the previous HR estimate is selected.

(31) $$\hat{hr}=\{e_{\overset{˚}{c}}^{\overset{˚}{n}};\;\;\underset{\overset{˚}{c},\overset{˚}{n}}{\mathrm{argmin}}\;\;|e_{c}^{n}-{\hat{hr}}^{prv}|\}.$$

The proposed spectral peak pruning routing is summarized in Algorithm 1.
**Algorithm 1:** Spectral-Based Peak Selection for Heart-Rate Calculation
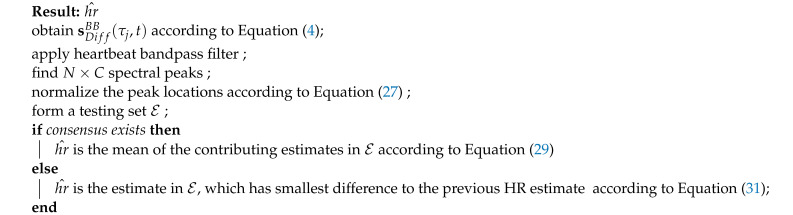


### 2.5. Evaluation Metrics

**Figure 6:**
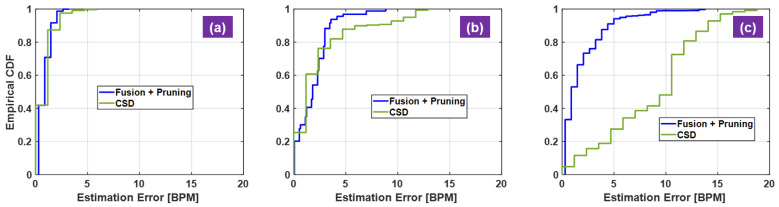
Heart-rate (HR) estimation error analysis from different poses. Blue curve denotes the proposed method using sensor fusion with spectral peak pruning algorithm and green curve the complex signal demodulation method (CSD). (**a**) stable seating; (**b**) free-style seating; (**c**) standing.

**Figure 7:**
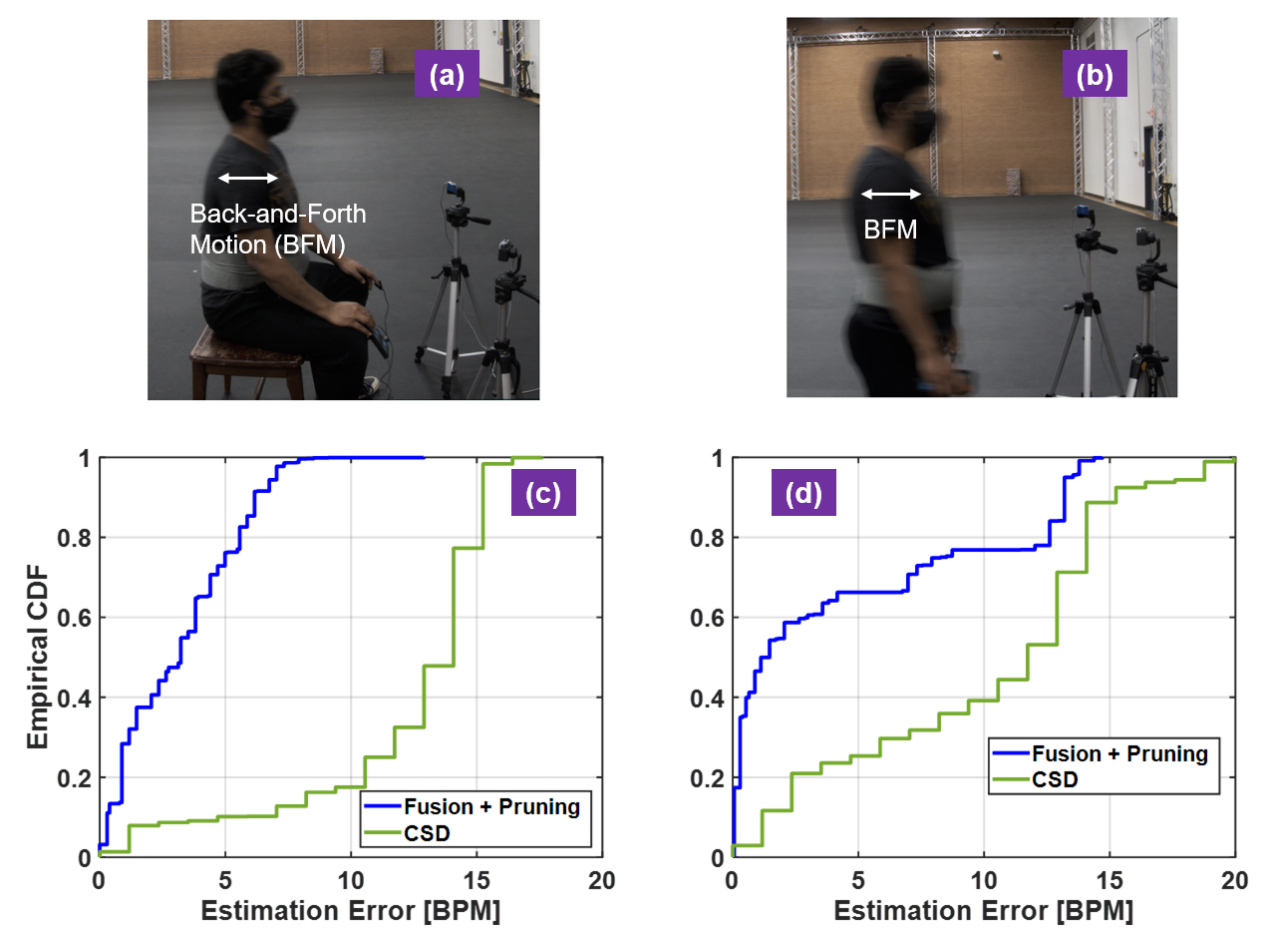
HR estimation error analysis for free-style seating and standing with back-and-forth motions (BFM). (**a**) experiment scene of free-style seating with BFM; (**b**) experiment scene of standing with BFM; (**c**) estimation CDF of free-style seating with BFM; (**d**) HR estimation error CDF of standing with BFM.

**Figure 8:**
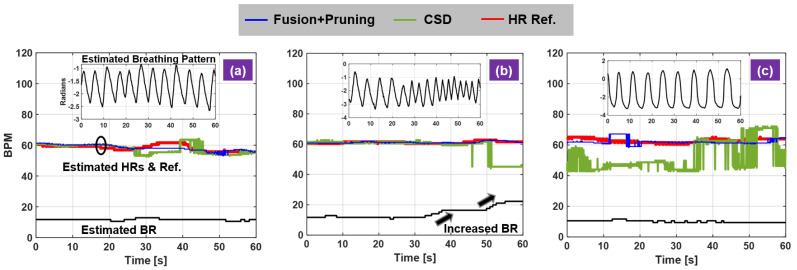
Investigation of dynamic breathing profiles on HR monitoring. (**a**) consistent shallow breathing; (**b**) varying regular breathing; (**c**) deep breathing.

**Figure 9:**
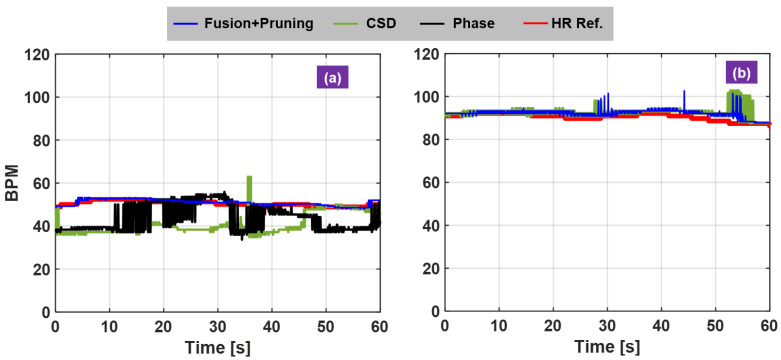
Investigation of heartbeat dynamics on HR monitoring. (**a**) low resting HR; (**b**) high resting HR.

Visual inspection and quantitative measures of the experimental results are used to justify the proposed radar algorithm. The RF waveform front single sensor and dual-sensor are compared to show the effect of RF fusion for motion cancellation (Figure 5a–c). Inspection of the corresponding the vital-sign spectra indicates the HR recovery performance (Figure 5d–i). For quantitative measures, HR error statistics are computed and displayed in terms of the empirical cumulative distribution function (CDF) of HR estimation errors (Table 1). The calculated empirical CDFs is used to compare different algorithms (Figure 6 and Figure 7). For visual inspection, the HR calculated with the proposed radar signal processing is compared against the reference HR from the acquired PPG signals (Figure 8 and Figure 9).

## 3. Motion-Tolerance Demonstrations

Two sets of studies are conducted for HR estimation error analysis in the presence of motion artifacts. The involuntary body motions from various poses and controlled BFMs are examined separately. These motion-tolerant experiments are repeated on three human subjects. The corresponding HR estimation error statistics for each subject and each case are summarized in Table 1 in terms of root mean squared error (RMSE) and standard deviation (STD), RMSE±STD. The HR results are generated with a 20 s sliding window with one sample increment. The number of equally spaced frequency intervals is chosen to be N=3 and up to 3rd heartbeat harmonics are used. The graphical representation of HR estimation performance of the subject 1 is displayed in Figure 6 and Figure 7 as an example.

The empirical CDFs of HR estimation error from stable seating, free-style seating and standing are shown in Figure 6. The proposed method includes active motion cancellation to recover meaningful spectrum at the frequency of interest and consequently the spectral peak pruning routine for consistent rate calculation. The complex signal demodulation method (CSD) [40,41] is added to the HR estimation performance comparison, in which only one sensor data from the left side is used and the dominant spectral peak in the heartbeat frequency region is selected to calculate HR. Three datasets, five minutes each, are used to produce the results in Figure 6a–c. From case in Figure 1a to case in Figure 6c, the stability of human body decreases, and the prolonged recording time captures realistic motion interference. The CSD performance significantly degrades from stable seating to standing as the human body stability decreases. On the other hand, the proposed method only degrades mildly in these cases. The performance gap between the proposed method and the CSD is largest in the standing example while their performance is almost comparable in sable seating example.

Controlled BFMs were introduced to the second set of experiments to challenge the motion-tolerant performance of the proposed algorithms. While the human subjects were seating (without back support) and standing with normal breathing, they were instructed to move back and forth slightly with maximum physical displacement about four centimeters as seen in Figure 7a,b. The accumulated BFM period is at least 60 s of the two minutes experiment time. Two datasets are used to produce Figure 7c,d. In these two challenging cases, the CSD method completely fails as the HR estimation is quite low with RMSE 12.70 BPM and 11.25 BPM. For example, 12% of the time the HR estimation error is less than 5 BPM in seating with BFMs and 22% of the time in standing with BFMs. The proposed method with active motion cancellation is effective with RMSE 3.89 BPM and 6.88 BPM. In addition, about 75% of the errors are within 5 BPM in seating with BFMs and about 67% in standing with BFMs.

## 4. Novel Breathing and Heartbeat Dynamics Analysis

Single radar platform with insufficient degree of freedoms (limited RF bandwidth and lack of spatial resolution) really limits the signal-processing capability for practical HR monitoring applications. The dual-radar fusion technique is demonstrated to be effective to handle HR monitoring in the presence of BFM to certain extent. However, still a few aspects are not well recognized and investigated. The dynamics of breathing activity makes a significant impact on HR monitoring and result in unreliable HR measurements. Three carefully designed experiments are demonstrated and compared side by side to illustrate this new insight on radar HR sensing. Three different breathing profiles are considered: consistent shallow breathing, varying breathing patterns from normal to fast breathing, consistent deep breathing. The test subject was seated in a stable chair with back support and instructed to perform these informed breathing activities while preventing any other body movements. The extracted breathing patterns from radar measurements are displayed in each subfigure. Their patterns match the pre-designed configuration. The breathing pattern in Figure 8a is regular shallow breathing with smaller amplitude, the breathing pattern in Figure 8b experiences a transition from slow to fast breathing as seen in the increased repetition cycle while the breathing pattern in Figure 8c has larger amplitude and increased breath interval corresponding to deep breathing profile. In the shallow breathing example, the CSD and the fusion-based method generates similar results. Both HR evolutions are consistent with the pulse reference. When breathing fast, the BR estimation increases and the strong breathing harmonics get closer to the fundamental HR. Thus, the CSD method starts deviating from the truth while the proposed method still provides accurate estimates. When breathing motion is exaggerated in the case of deep breathing, the conventional method fails to provide consistent results throughout the experiment. Robust measurement is achieved in the proposed method enabled by active motion cancellation in the RF stage. Note the exaggerated breathing motion is demonstrated as a source of RBMs that challenges the HR monitoring performance and validate our approach.

Another novelty of this study is the investigation of human subject dependent characteristics, the different resting HR among human subjects. HR statistics for normal healthy people is about 60–100 BPM. For robust radar algorithm it should work for most individuals. For example, resting HR for well-trained athletes can be well under 60 BPM and even close to 40 BPM while for some it can be close to 100 BPM. This is an important consideration, as it breaks the original basis of spectral-based HR calculation that HR and BR are further apart and thus separable. In this regard, people with lower resting HR are difficult to detect because the fundamental HR is inside the breathing harmonic frequency region. The following examples validate this new insight. Two test subjects were selected based on their resting HRs: one about 50 BPM and the other one about 90 BPM, both subjects were instructed to breath at 17–20 BPM range to ensure the ambiguity for HR detection for the subject with lower resting HR. For the low resting HR subject, the CSD method and phase-based method constantly underestimate HR due to stronger breathing harmonics however the proposed method can trace the HR over time (Figure 9a). On the contrary, for the high resting HR subject, both methods can perform HR monitoring by comparing against the pulse reference (Figure 9b).

## 5. Conclusions

A novel motion-tolerant non-contact HR estimation algorithm is presented and demonstrated with a dual-UWB impulse radar system. The active motion cancellation is achieved via direct RF signal fusion from the two radar sensors. The radar sensors are placed at chest height about 60 cm away at equal distance. They are pointing at different sides of the chest to create spatial diversity and differential measurements for enhanced HR detection when RBMs occur. A spectral-based HR peak pruning routing is proposed to deal with the ambiguity issue for detecting HR by exploiting higher-order spectral features of heartbeat. The proposed method is theoretically proved by an analytical analysis of the spectral representation of the differential complex baseband signal model. The developed algorithm is validated through a comprehensive experimental study for three human subjects. HR measurement performance are compared for three different poses, including stable seating, free-style seating and standing. A robustness test is conducted in the presence of BFMs, showing effectiveness of the proposed algorithm.

Additionally, two insights toward robust HR sensing using radar sensors are introduced and discussed. Radar sensors are motion-based measurement and thus they are sensitive to heartbeat and breathing motion. The latter one is stronger and always coupled with heartbeat. Naive spectral separation is not sufficient for differentiating the two activities, which is the basis for most popular methods for radar. In this regard, three different breathing profiles are investigated from suppressed breathing, fast varying breathing and deep breathing, in which the conventional radar signal processing behaves differently and fails to detect the fundamental HR due to stronger breathing motion and nearby breathing harmonics. A robust radar approach for HR is necessitated to monitor people with different resting HR. The HR diversity comes from the fact that people have different physical conditions, emotional states, lifestyles and more. It is shown to be difficult for the conventional radar signal processing to track HR for low resting HR human subject due to the reduced spectral gap between breathing and heartbeat. The proposed theory provides a new perspective on addressing these issues.

## Figures and Tables

**Table 1:** Summary of HR Estimation Error Statistics (BPM) in Various Scenarios.

	Subject 1		Subject 2		Subject 3	
Algorithms	Proposed (RMSE, STD)	CSD	Proposed	CSD	Proposed	CSD
Stable Seating	1.05, 0.59	1.27, 0.92	1.38, 0.90	1.25, 0.88	1.55, 1.03	2.34, 1.03
Free-Style Seating	2.49, 1.60	3.98, 3.13	2.66, 1.80	4.59, 3.71	2.72, 2.02	3.68, 3.55
Standing	2.90, 2.20	9.71, 4.53	2.27, 1.80	10.30, 4.44	3.23, 1.98	9.08, 4.62
Seating + BFMs	3.89, 2.26	12.70, 4.72	4.16, 2.51	13.44, 4.56	3.74, 3.45	8.21, 5.02
Standing + BFMs	6.88, 5.26	11.25, 5.39	8.22, 4.98	15.21, 6.11	7.55, 6.22	13.46, 5.87

## Data Availability

The data that support the findings of this study are available from the corresponding authors upon reasonable request.

## Abbreviations

The following abbreviations are used in this manuscript:

VSM	Vital-sign monitoring
RBM	Random body movement
HR	Heart-rate
UWB	Ultra-wideband
BR	Breathing rate
PPG	Photoplethysmography
GHz	Gigahertz
BFM	Back-and-forth motion
RF	Radio frequency
2-D	2-dimensional
BPM	Beats per minute
FT	Fourier transform
CDF	Cumulative distribution function
RMSE	Root mean squared error
STD	Standard deviation
CSD	Complex signal demodulation

## Appendix A. Derivation of $s_{L}^{BB}\left(\tau ,t\right)$ in Equation (15)

The detailed derivation of Equation (15) is provided. Equation (19) can be deducted in the similar way. The goal of spectral analysis is to inspect BFM frequency $f_{R}$, breathing frequency $f_{B}$, heartbeat frequency $f_{H}$ and associated harmonics. This can be achieved by taking FT of $s_{L}^{BB}\left(\tau ,t\right)$,

(A1) $$s_{L}^{BB}\left(\tau ,f\right)=\int_{-\infty }^{\infty }dt\;\;s_{L}^{BB}\left(\tau ,t\right)e^{-j2\pi ft},$$

Instead of directly working on Equation (A1), it is often more useful to work on the 2-D FT of $s_{L}^{BB}\left(\tau ,t\right)$, $s_{L}^{BB}\left(\nu ,f\right)$, to get $s_{L}^{BB}\left(\tau ,f\right)$,

(A2) $$s_{L}^{BB}\left(\tau ,f\right)=\int_{-\infty }^{\infty }d\nu \;\;s_{L}^{BB}\left(\nu ,f\right)e^{j2\pi \nu \tau },.$$

The 2-D FT $s_{L}^{BB}\left(\nu ,f\right)$ is first derived,

(A3)sLBB(ν,f)=∫−∞∞∫−∞∞dtdτsLBB(τ,t)e−j2πfte−j2πντ=∫−∞∞dte−j2πft∫−∞∞dτsLBB(τ,t)e−j2πντ=∫−∞∞dte−j2πft∫−∞∞dτATp(τ−τD,L(t))e−j2π(fc+ν)τ(A4)        =∫−∞∞dte−j2πftATp(ν)e−2πντD,L(t)(A5)        =ATp(ν)e−j4πντ0∫−∞∞dte−j2πfte−j4πνR(t)ce−j4πνB(t)ce−j4πνH(t)c,

where change of integration variable is applied from Equations (A3) and (A4). $\tau_{0}$ is the time-delay associated with the target distance. The time-varying delay, $\tau_{D,L}\left(t\right)$, is defined in Equation (9). By invoking the expansion of a series of Bessel functions [38] on the last three terms in Equation (A5), $s_{L}^{BB}\left(\nu ,f\right)$ can be written as,

(A6)sLBB(ν,f)=ATp(ν)e−j4πντ0∫−∞∞dte−j2πft×∑k=−∞∞Jk4πνARce−j2πkfRt∑l=−∞∞Jk4πνABce−j2πlfBt∑q=−∞∞Jk4πνAHce−j2πqfHt(A7)      =∑k=−∞∞∑l=−∞∞∑q=−∞∞ATp(ν)e−j4πντ0Jk4πνARcJl4πνABcJq4πνAHc      ×∫−∞∞dte−j2π(f+kfR+lfB+qfH)t.

Plugging Equation (A7) into (A2), the spectral representation of interest is obtained and consequently the derivation of Equation (15) completes.
